# Combined application of *Ascophyllum nodosum* extract and chitosan synergistically activates host-defense of peas against powdery mildew

**DOI:** 10.1186/s12870-020-2287-8

**Published:** 2020-03-12

**Authors:** Jai Singh Patel, Vinodkumar Selvaraj, Lokanadha Rao Gunupuru, Pramod Kumar Rathor, Balakrishnan Prithiviraj

**Affiliations:** Department of Plant Food and Environmental Sciences, Dalhousie University, Nova Scotia, Canada

**Keywords:** Plant-microbe interaction, Seaweed extract, Plant immunity, Sustainable agriculture

## Abstract

**Background:**

Powdery mildew (PM) is an important disease of pea that reduce yield. *Ascophyllum nodosum* extract (ANE) and chitosan (CHT) are biostimulants used to improve plant health. Efficacy of ANE and CHT was assessed individually and in combination against pea powdery mildew.

**Results:**

Combined applications of ANE and CHT had a significant inhibitory effect on pathogen development and it reduced disease severity to 35%, as compared to control (90.5%). The combination of ANE and CHT enhanced the activity of plant defense enzymes; phenylalanine ammonia lyases (PAL), peroxidase (PO) and production of reactive oxygen species (ROS) and hydrogen peroxide (H_2_O_2_). Further, the treatment increased the expression of a number of plant defense genes in jasmonic acid (JA) signaling pathway such as *LOX1* and *COI* and salicylic acid (SA)-mediated signaling pathway such as *NPR1* and *PR1*. Other genes involved in defense mechanisms like *NADPH oxidase* and *C4H* were also upregulated by the combination treatment*.*

**Conclusion:**

The combination of ANE and CHT suppresses pea powdery mildew largely by modulating JA and SA-mediated signaling pathways.

## Background

Powdery mildew (PM) of pea (*Pisum sativum* L.) is caused by an obligate, parasitic fungus *Erysiphe pisi*. Powdery mildew infects all above-ground parts of pea plant, which causes significant negative effects on productivity, including a reduction in the number of pods and seeds [1] The disease was reported to cause 25–50% yield loss [2, 3]. *Erisyphe pisi* propagates through the production of ascospores and conidia [4]. Both conidia and ascospores germinate on pea plant, develop appressoria on the surface and infect epidermis [5, 6].

Indiscriminate use of pesticides has resulted in the development of resistance in a number of common pathogens, as well as losses of beneficial microflora and fauna. Furthermore, there is an increasing demand for pesticide-free food amongst discerning consumers which requires the development of eco-friendly plant protection practices. The use of seaweed-derived products in agriculture has been steadily increasing in the recent years. *Ascophyllum nodosum* is an intertidal, brown alga found around the north Atlantic Ocean and the northwestern coast of Europe [7]. *A. nodosum* biomass is used to produce one of the most commonly studied seaweed-based biostimulants [8–10]. Commercial extracts of the brown alga have been reported to enhance plant growth as well as to promote the growth of beneficial soil microbes and induce plant resistance against biotic and abiotic stresses [9, 11]. *Ascophyllum nodosum* extracts (ANE) have been reported to suppress disease incidence and the growth of various pathogens including *Alternaria cucumerinum*, *Didymella applanata*, *Fusarium oxysporum*, *Botrytis cinerea* [12, 13], *Colletorichum lagenarium* [14], *Phytophthora capsica* [15] and *Verticillium* sp. [16]. The macro- and micronutrients, as well as the phyco-elicitors (compounds similar to plant hormones such as cytokinins, auxins and abscisic acid (ABA)-like substances) that are present in the macroalgal extracts were reported to have a beneficial effect on plant cellular metabolism, leading to enhanced crop growth and yield [17, 18]. Biologically active auxin-like compounds, and indole acetic acid (IAA), have also been reported in the alkaline hydrolysates of *A. nodosum.* In addition to these compounds, *A. nodosum* extracts also contains unique polysaccharides such as laminarin, fucoidan, and alginic acids [9, 19]. Chitosan (CHT) is a naturally occurring biopolymer (a derivative of chitin) that was shown to elicit plant defense mechanisms against a number of pathogens [20, 21]. Chitosan treatment increased the synthesis of pathogenesis-related (PR) proteins, proteinase inhibitors, phytoalexins as well as callus formation and lignin synthesis. Root injection of chitosan in date palm (*Phoenix dactylifera* L.) elicited polyphenol oxidase (PPO) and peroxidase (PO) activities, resulting in increased levels of phenolic compounds [22]. Several other studies reported the use of chitosan against plant pathogens such as *Erysiphe* sp. and *Blumeria graminis* f. sp. *hordei* [23–25].

Several studies have shown ANE and CHT trigger plant defense responses by inducing the expression PAL, PO and H_2_O_2_. Foliar spray of ANE elicited activity of defense-related enzymes, including peroxidase (PO), polyphenol oxidase (PPO), phenylalanine ammonia lyase (PAL) and chitinase in carrot [12] and cucumber [13]. Application of ANE on pepper plants resulted in multifold increases in peroxidase activity and phytoalexin synthesis [15]. Incorporation of ANE to planting medium resulted in the accumulation of higher concentration phenolics in pepper [16]. Similar results were reported with chitosan application. Spray treatment of okra plants with chitosan increased the total phenolic content and increased polyphenol oxidase, peroxidase, chitinase, and β-1,3-glucanase [26].

Earlier reports suggest ANE and CHT protect plant against pathogen by eliciting systemic resistance. ANE applications induced systemic resistance, largely through the jasmonic acid-dependent pathway [27]. In contrast, chitosan application elicited systemic acquired resistance, which is a salicylic acid mediated pathway [28]. This study focused on the effect of combined application of ANE and CHT on the development of PM, and possible mechanism(s) of action that leads to increased resistance against PM in pea.

## Results

### ANE and CHT reduce powdery mildew disease severity in pea

**Fig. 1:**
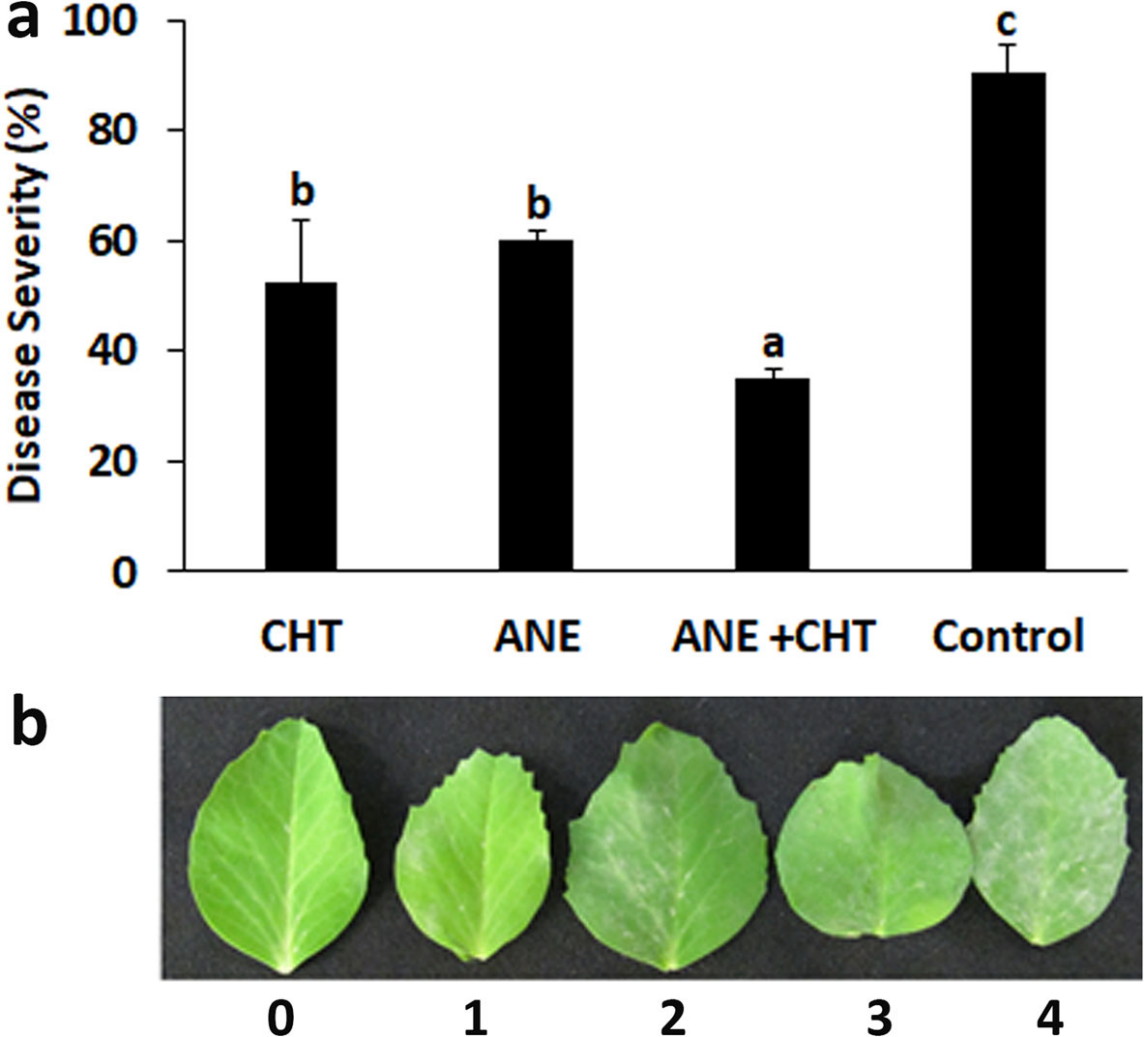
Suppression of powdery mildew by *Ascophyllum nodosum* extract and chitosan. (CHT = Chitosan 100 ppm, ANE = *A. nodosum* Extract 0.015%). The data was recorded 15 days after pathogen inoculation. 10 replicates with 12 plants in each replicate were used in the experiment. Error bars represent SD (Standard Deviation). Bars with different letters are significantly different (*P* ≤ 0.05; Duncan’s multiple range test)

**Fig. 2:**
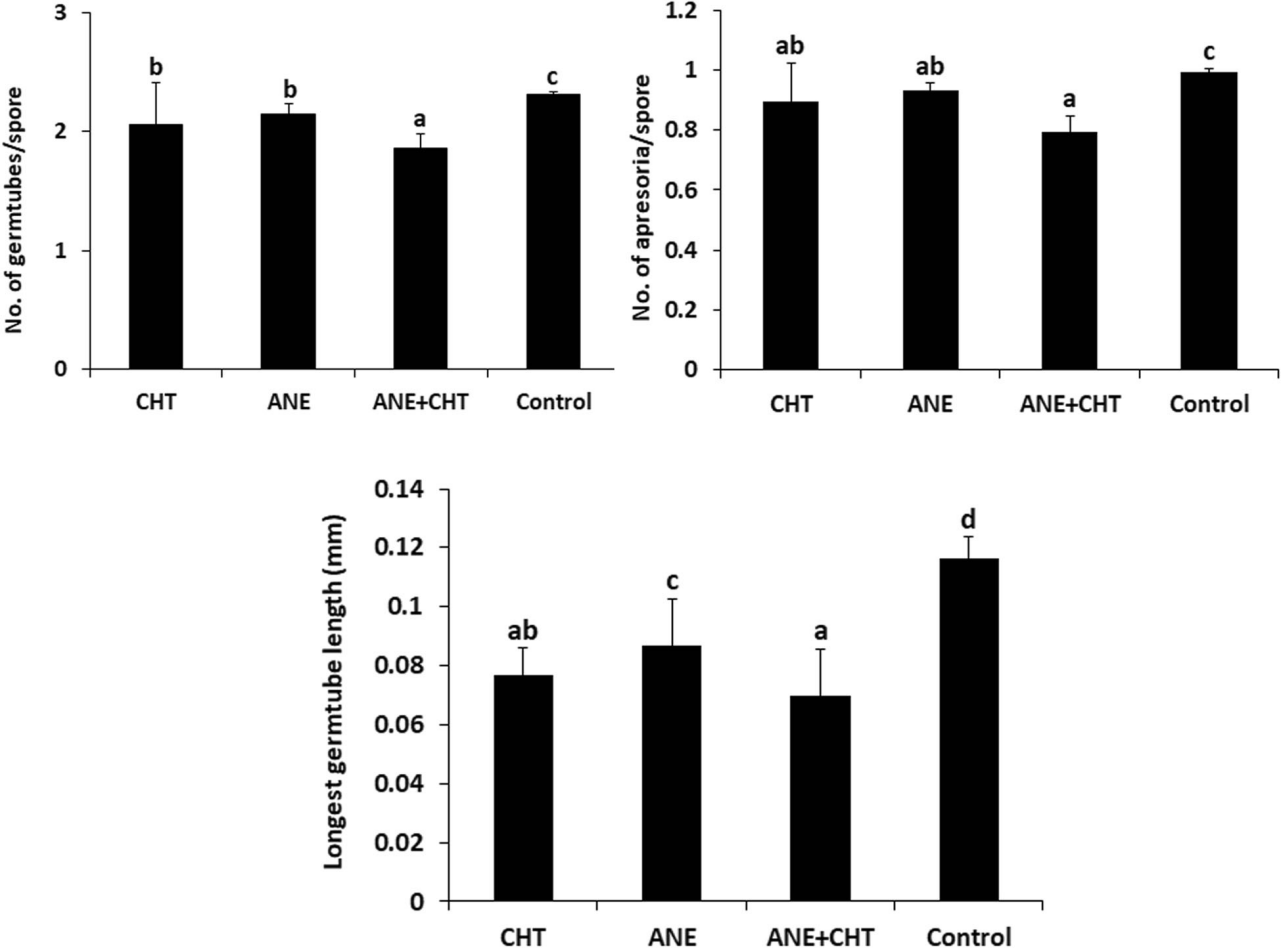
Effects of *Ascophyllum nodosum* extract and chitosan on powdery mildew conidial germination. **a** number of germ tubes/spore, **b** number of appressoria/spore, **c** Average length of germ-tube (mm). (CHT = Chitosan 100 ppm, ANE = *A. nodosum* extract 0.015%, Control = Sterilized distilled water). Randomly five leaves were used per plant from 12 plants with three replicates, after pathogen inoculation. Error bars represent SD (Standard Deviation). Bars with different letters are significantly different (*P* ≤ 0.05; Duncan’s multiple range test)

**Fig. 3:**
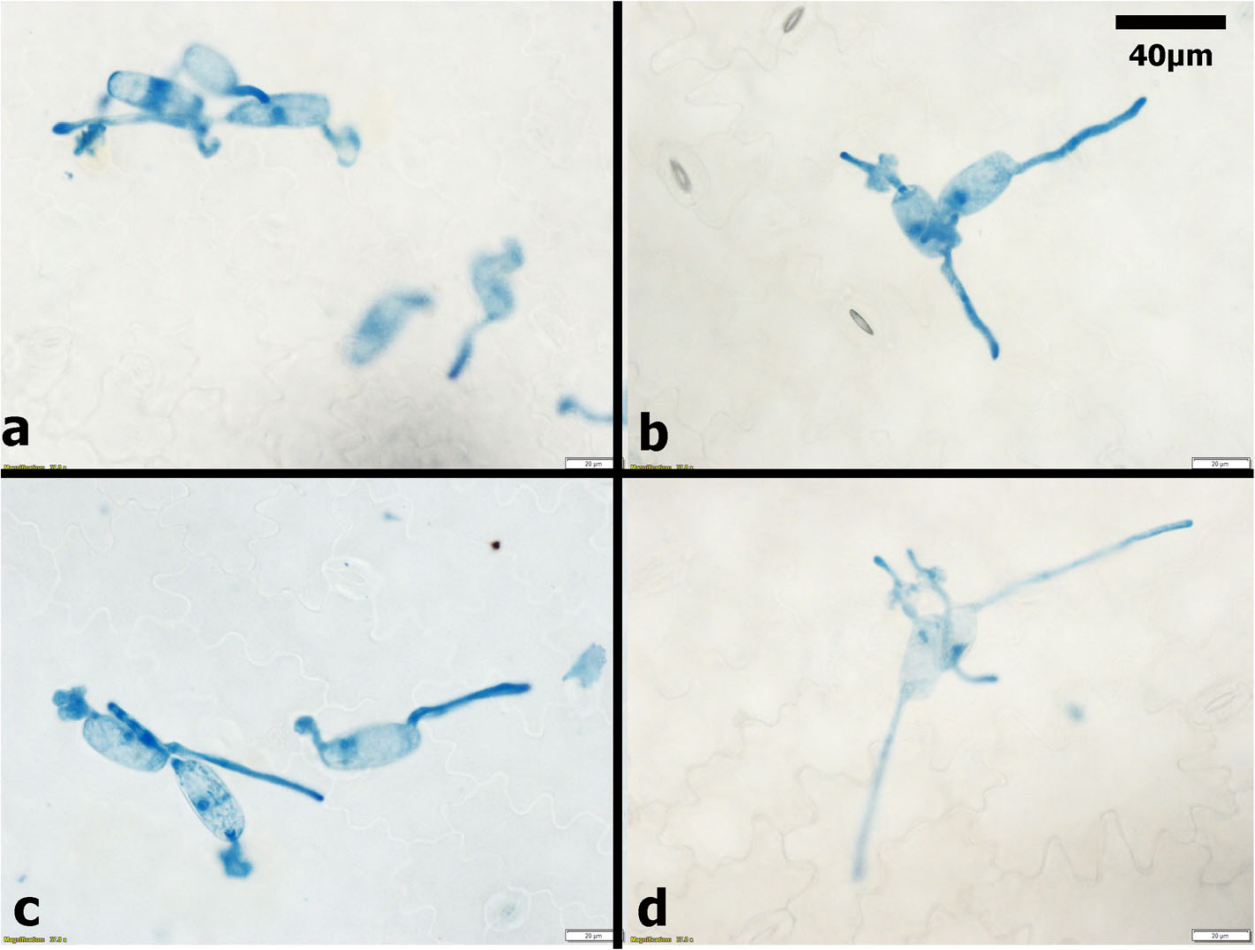
*Ascophyllum nodosum* extract and chitosan inhibited conidial germination of powdery mildew. **a** CHT (chitosan 100 ppm) **b** ANE (A. nodosum extract 0.015%) **c** ANE + CHT **d** Control (sterile distilled water)

**Fig. 4:**
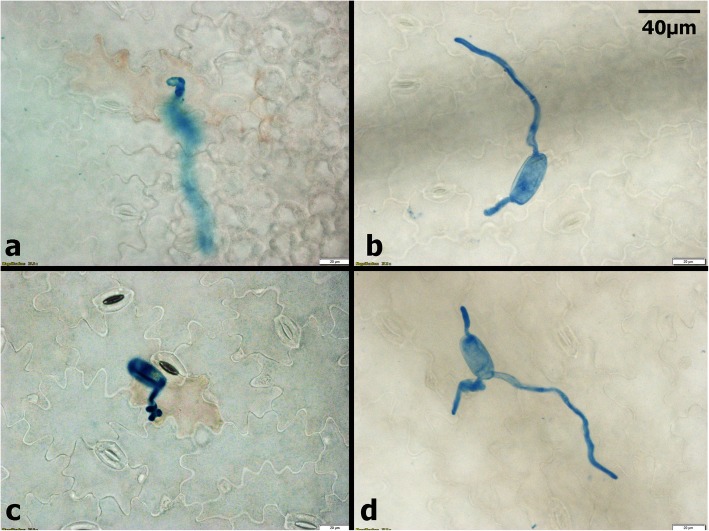
*Ascophyllum nodosum* extract and chitosan induced hypersensitive response against powdery mildew pathogen. ** a** CHT (chitosan 100 ppm) **b** ANE (A. nodosum extract 0.015%) **c** ANE + CHT **d** Control (sterile distilled water)

Pea seedlings (21 days post-planting) sprayed with ANE and CHT either alone or in combination exhibited enhanced resistance to the powdery mildew. Disease severity in all treatments was significantly (*P ≤ 0.05*) lower compared to the control (sprayed with sterilized distilled water). The percent disease incidence (PDI)) in treatments were 35% in ANE + CHT, 60% in ANE, and 52.5% in CHT- 52.5%, as compared to 90.5% in the control (Fig. 1). The combined application also inhibited pathogen establishment by supressing the development of germ tubes to 0.45/spore, appressoria to 0.2/spore and germ tube length to 0.046 mm, compared to the control (Figs. 2 and 3). Moreover, browning of the plant cells adjacent to the germinating spores was also observed (Fig. 4). This indicated a hypersensitive response, triggered by the pathogen challenge, in CHT and ANE + CHT treatments.

### ANE and CHT synergistically induce plant defense responses

**Fig. 5:**
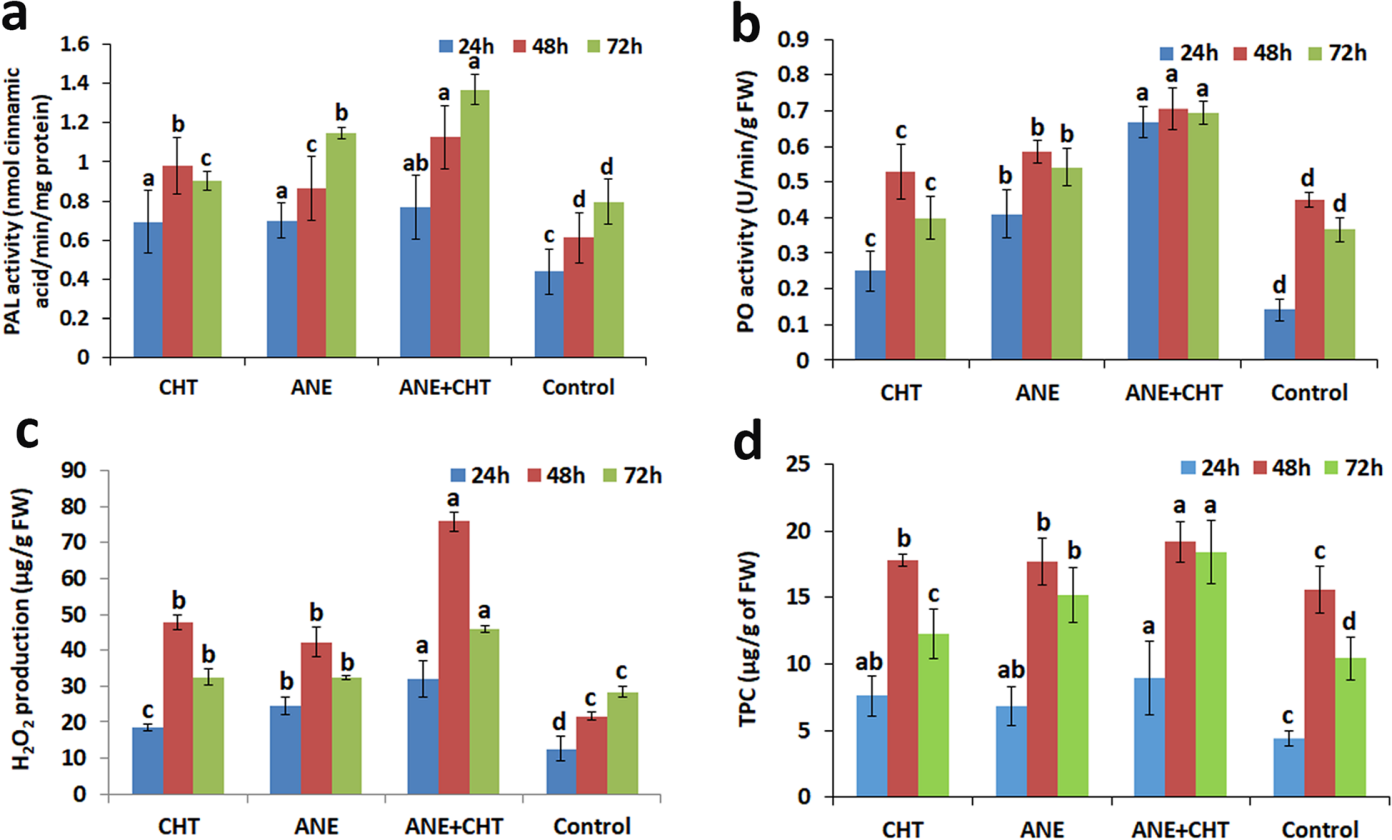
Effects of *Ascophyllum nodosum* extract and chitosan on reactive oxygen species production and antioxidant enzymes in pea leaves. **a** Phenylalanine ammonia lyases activity, **b** Peroxidase activity, **c** H_2_O_2_ production, and **d** Total phenolic content. (CHT = Chitosan (100 ppm), and ANE = *A. nodosum* extract 0.015%, Control = Sterilized distilled water). Samples were collected in triplicate from 12 plants in each replicate. Error bars represents SD (Standard deviation). Bars with different letters are significantly different (*P* ≤ 0.05; Duncan’s multiple range test)

ANE and CHT both when applied alone or in combination induced plant defense related enzymes like phenyl alanine ammonia lyase (PAL) and peroxidase and as well as increased the concentration of total phenolics. The phenyl alanine ammonia lyase (PAL) activity increased gradually 24 h after treatment and was the highest at 72 h post treatment. Plants treated with ANE + CHT exhibited the highest PAL activity (1.369 nmol cinnamic acid/min/mg protein), followed by the ANE alone (1.14 nmol cinnamic acid/min/mg protein) while CHT alone (0.90 nmol cinnamic acid/min/mg protein) had a higher PAL activity as compared to the control, and all treatments were statistically significant at *P ≤ 0.05* (Fig. 5a). Peroxidase activity gradually increased after 24 h, and at 0.70 Unit/min/g fresh weight (FW) was the highest in the ANE + CHT treatment after 48 h. After 72 h of pathogen challenge, PO activity dropped in the combined treatment to 0.01 U/min/g FW. The ANE + CHT treatment therefore outperformed all other treatments. Interestingly, PAL activity in plants treated with ANE + CHT was stable, did not vary over time (Fig. 5b). The highest H_2_O_2_ production (75.73 μg/g of FW) was observed in ANE + CHT, 48 h after the treatment, and the activity decreased sharply at 72 h. The H_2_O_2_ concentration in CHT (47.7 μg/g of FW) and ANE (42.21 μg/g of FW) treated plants were similar (Fig. 5c). Concentration of total Phenolics in pea plants treated with the combination treatment were 8.89, 19.18 and 18.38 μg/g of FW 24, 48 and 72 h, respectively and was significantly higher (*P ≤ 0.05*), as compared with all other treatments (Fig. 5d). Notably, the accumulation of TPC in plants from the ANE + CHT treatment was stable and did not decline significantly at 72 h.

### ANE and CHT enhanced the accumulation of transcripts involved in defense response mechanisms

**Fig. 6:**
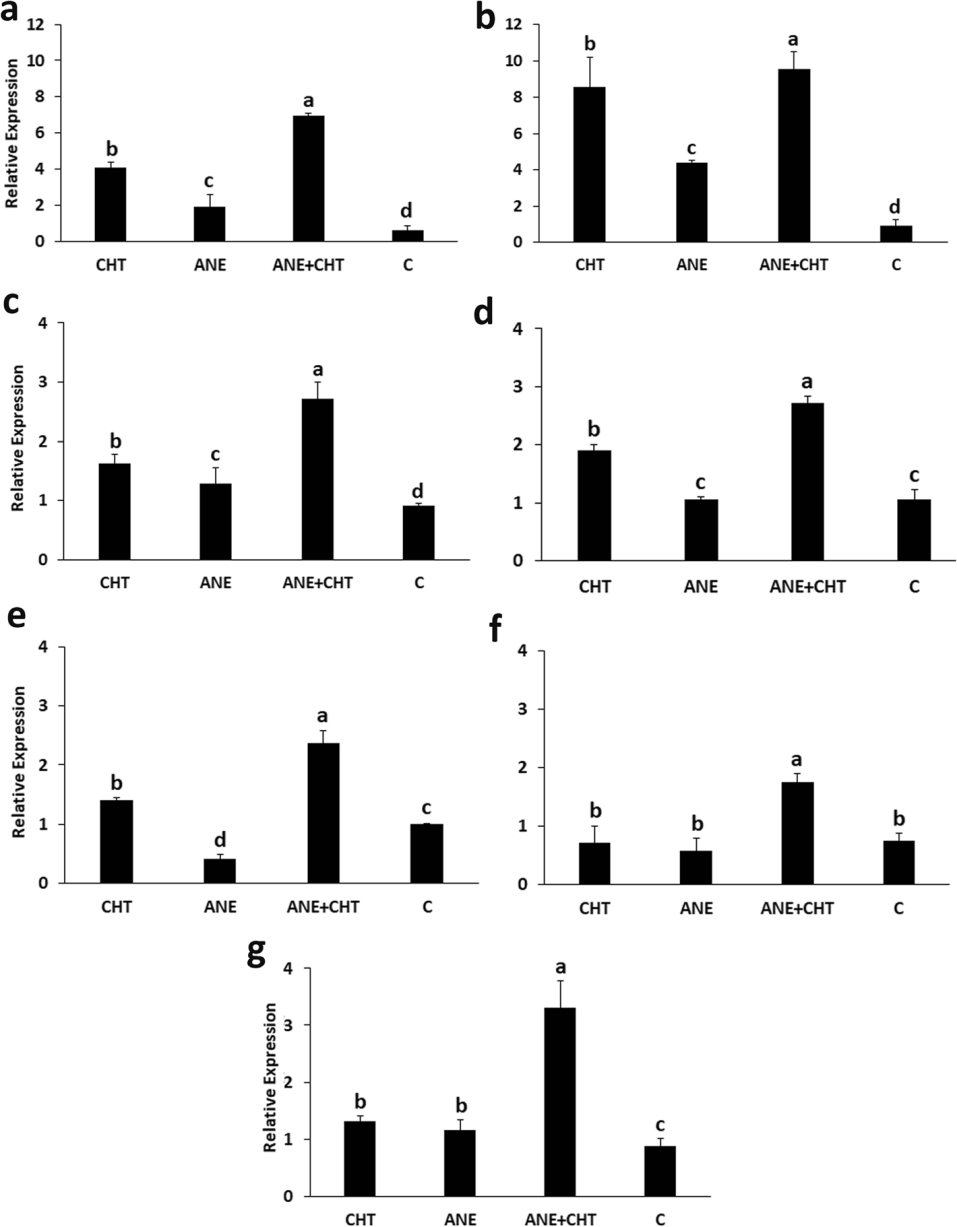
Effect of *Ascophyllum nodosum* extract and chitosan on the transcription of defense response genes **a***NPR*1, **b***COI*, **c***LOX,***d***PR1,***e***PDF1.2,***f***NADPH Oxidase*, and **g***C4H*. (CHT = Chitosan 100 ppm, and ANE = *Ascophyllum nodosum* Extract 0.015%, Control = Sterilized distilled water). Triplicate leaf tissue samples from 12 plants in each replicate were used in the experiment. Bars with different letters are significantly different (*P* ≤ 0.05; Duncan’s multiple range test)

Pea plants treated with ANE + CHT had the highest transcript abundance of defense response genes (Fig. 6). *NPR1* and *PR1* gene transcripts were relatively higher, 10.41 fold and 2.57 fold increases respectively, as compared to the un-treated control. This indicated the activation of SA-mediated signalling. Similarly, the combined treatment also increased transcripts of JA-mediated signalling genes, *LOX* (11.09 fold), *COI* (2.96 fold) and *PDF1.2* (2.38 fold) as well as cinnamic acid-4-hydroxylase (*C4H*) (3.72 fold) gene.

## Discussion

In the present study, the combined treatment of ANE + CHT outperformed other treatments (ANE or CHT alone) in suppressing powdery mildew through the induction of defense mechanisms in pea plant. Commercial extracts of *A. nodosum* have been reported to be efficient in mitigating several biotic and abiotic stresses in plants. ANE also promote plant growth while polysaccharides and oligosaccharides present in the extract are potent elicitors (phyco-elicitors) of plant defense responses [29].

Anti-fungal activity of ANE have been well documented against various plant pathogens [13]. Similarly, the anti-fungal activity of chitosan was reported [10]. Foliar spray of chitosan reduced powdery mildew in plants such as okra [26] barley [23] and grapevine [25]. Moreover, chitosan exhibited direct anti-fungal activity inhibiting spore germination of a number of plant pathogens. Chitin oligosaccharides inhibited spore germination and resulted in pore-formation and cellular leakage in the growing hyphae of *Fusarium* sp. [30]. Mycelial growth and spore germination of *Colletotrichum gloeosporioides* was also inhibited by chitosan. In addition, the treatment resulted in structural degradation of the hyphae [31]. However, the efficacy of the combined effect of ANE and CHT on PM infection have not been reported. In present study the combined application of two biostimulants resulted in a better disease suppression as compared to individual treatments. Furthermore, the combined treatment reduced the number of germ tubes/conidia, the number of appressoria/conidia and germ-tube length.

*A. nodosum* extract-treatment increased transcripts of pathogenesis-related protein I (*PR-I*), lipid transfer protein (*LTP*), phenylalanine ammonia lyases (*PAL*), *NPR-I, PR-5, WRKY30*, and *CYP71A12* [12, 13]. Previous reports showed that ANE treatment induced resistance in *Arabidopsis* mutants, deficient in salicylic acid (*NahG* and *ics1*), and they failed to do so in *jar1* mutants. These results indicated that ANE activates the JA-dependent defense mechanism in plants [27]. Chitosan application induced the expression of PR protein such as chitinase and chitosanase, enzymes involved in the degradation of the cell wall of pathogens [32]. CHT applications induced key genes in both jasmonic acid [33] and salicylic acid [34] signaling pathways. Applications of CHT in rice plants induced key components of the octa-decanoic pathway such as 12-oxo-phytodienoic acid (OPDA) and jasmonic acid [35]. Other studies reported increased production of jasmonic acid (JA) upon CHT treatment in plants such as tomato [36], rice [35] and rape seed [37]. In contrast, a few studies reported increased production of salicylic acid (SA) [28, 38]. The interaction between JA and SA defense pathway is complex and is likely to have evolved to fine-tune defense against continuously changing pathogen pressure. Both positive and negative interactions between the JA and SA mechanisms have been suggested. However, most of the interaction seem to be antagonistic in nature, where one suppresses the expression of the other. JA-dependent PR proteins including plant defensin 1.2 (*PDF1.2*), thionin2.1 (*THI2.1*), hevein like protein (*HEL*), and chitinaseB (*CHIB*) are commonly used to monitor JA-dependent defense responses [39]. *NPR1* is a central regulator of plant defense responses and connects both SAR (Systemic Acquired Resistance) and ISR (Induced Systemic Resistance) [40, 41]. Furthermore, this gene has been reported for effectiveness against a large variety of organisms in SAR response [42].

The combined applications of ANE and CHT, resulted in the higher expression of of SA-mediated *NPR1, PR1,* as well as that of the JA-mediated *LOX, COI* and *PDF1.2* genes*.* These observations suggest the possibility of SA and JA mediated signal resulting in suppression of PM infection. In this study, H_2_O_2_ production was significantly (*P ≤ 0.05*) higher in ANE + CHT treatment. The increase in transcripts of *NADPH*-oxidase may be correlated with ROS production [43]. Browning of host cells around the germinating conidia ot the site of PM infection indicated the possibility of hypersensitive response in plants treated with ANE + CHT. Further study using genomics, metabolomics or protemics approaches will contribute to the characterization of the mode of action of combination of ANE and CHT.

## Conclusion

The combined application of ANE and CHT exhibited higher anti-fungal activity as compared to individual treatment. The components triggered the production of plant defense-related enzymes and metabolites. Furthermore, the combined treatment also induced transcripts of the SA and JA-dependent plant defense genes, which resulted in reduction of powdery mildew infection.

## Methods

### Preparation of pea plants and pathogen inoculum

Pea plant (cv. Sabre, Veseys Seed Canada) was grown in pots containing Promix® (Premier Tech, QC, Canada). Plants were grown in greenhouse maintianed at 16:8 h light:dark cycle, at 21 ± 2 °C. Pathogen inoculum was collected from naturally infected pea plants in the field (Chef’s Garden, Dalhousie University, Truro, Canada). The pathogen was identified by morphological characterization.

### Treatments and inoculation of pathogen

Pea plants were spray treated with ANE - 0.015% (Acadian Seaplants Limited, chemical composition of ANE was reported earlier [44]) and chitosan (CHT) – 100 ppm (Sigma Aldrich®), individually and in combination. Plant was sprayed until drip. Control plants were sprayed with sterile distilled water. Treated plant was inoculated with *Erysiphe pisi* after 48 h following protocol described previoulsy [45]. The experiment was repeated three times with 10 replicates per treatments and 12 plants per replicate.

### Efficiency of ANE and CHT in the suppression of PM disease development

Leaf samples were collected at 24 h and 48 h post-inoculation to assess the efficiency of the treatment on conidial germination. The leaf was de-chlorophyllized with ethanol:acetic acid solution (3:1). The leaves were stained with lactophenol cotton blue (Merck, Germany). The number of germ-tubes/conidia, germ-tube length, number of appressoria/conidia were observed. Fifty spores were counted inr each treatment and mean value was obtained. Disease severity was observed 15 days, post inoculation, following 0–4 scale [46], where 0 = no visible sign of infection, 1 = 25% leaf area infected, 2 = 50% leaf area infected, 3 = 75% leaf area infected and 4 = 100% leaf are infected with freely sporulating colonies (Fig. 3b). The percent disease index (PDI) was calculated following formula given below.

$$ \mathrm{PDI}=\frac{\mathrm{Sum}\ \mathrm{of}\ \mathrm{ratings}\ \left(0-4\right)\ \mathrm{x}\ 100}{\mathrm{Maximum}\ \mathrm{possible}\ \mathrm{score}\ \mathrm{x}\ \mathrm{Total}\ \mathrm{no}.\mathrm{of}\ \mathrm{leaves}\ \mathrm{examined}} $$

### Effect of ANE and CHT on induction of plant defense response

Leaf samples were collected from PM-inoculated plants at 24 h, 48 h and 72 h post inoculation. Each treatment had 12 plants per replicate and the experiment was repeated twice.

### Phenylalanine ammonia lyases (PAL)

Leaf sample (0.5 g per treatment) was ground in 4 mL borate buffer (pH 8.7; 4 °C) and centrifuged at 13,000 rpm for 15 min at 4 °C. The supernatant was used as the enzyme source in the reaction mixture. The reaction mixture containing 0.2 mL enzyme extract, 1.3 mL distilled water and 1.0 mL phenylalanine (0.1 M) was incubated at 32 °C for 30–60 min and the reaction was stopped by addition of 0.5 mL of 1 M trichloroacetic acid. PAL activity was measured following the formation of trans-cinnamic acid at 290 nm as described [47] and expressed in terms of nmol trans-cinnamic acid per minute per milligram of fresh weight (FW).

### Peroxidase (PO)

The peroxidase (PO) assay was performed following published protocol [48]. Leaf tissue (1 g) was extracted in 4 mL 0.1 M phosphate buffer (pH 7.0). The supernatant was used as the enzyme source and the reaction mixture consisted of; 0.1 mL enzyme extract, 2.8 mL of 0.1 M phosphate buffer (pH 7.0), 0.05 mL of 0.018 M guaiacol and 100 μL of 1% H_2_O_2_. Absorbance of the reaction mixture were recorded using a spectrophotometer (Cytation 5 - BioTek, USA) (λ = 420 nm) every 30 s for 3 minutes (6 absorbance values per reaction mixture). Enzyme activity was expressed as the change in OD per minute per gram of FW.

### Hydrogen peroxide (H_2_O_2_)

For quantification of H_2_O_2_, 0.1 g leaf sample was ground in 2.0 mL of 0.1% (w/v) trichloroacetic acid in an ice bath. The extract was centrifuged at 12,000 g for 10 min. The reaction mixture contained 0.5 mL of the supernatant, 10 mM potassium phosphate buffer (pH 7.0) and 1 mL of 1 M potassium iodide. The mixture was incubated at room temperature for 5 min and absorbance measured using a spectrophotometer (Cytation 5 - BioTek, USA) (λ = 390 nm) [49]. The amount of H_2_O_2_ was determined by comparing the results with a standard curve made with known concentrations of H_2_O_2_ and expressed in terms of nmol H_2_O_2_ g^− 1^ fresh weight (FW).

### Total phenolic content (TPC)

Total phenolic content (TPC) was quantified following the method described previously [50]. Leaf tissue (1 g) was extracted in 50% methanol. The supernatant was evaporated, dissolved in distilled water (1 mL) and analyzed with a spectrophotometer following the Folin-Ciocalteau method [51]. TPC was calculated in terms of gallic acid equivalents using a gallic acid standard curve.

### Effects of ANE and CHT on the defense related genes

The relative expression of *NPR1*, *LOX1, PDF1.2, NADPH Oxidase, C4H,* and *PR1* were determined using quantitative real-time PCR (qRT-PCR). Total RNA was extracted from 200 mg powdered leaf tissue using RNeasy plant mini-kit (QIAGEN) following the manufacturer’s protocol. Three leaf samples were collected from each of the 12 replicate plants and pooled together to obtain total RNA. The quantity and quality of the total RNA was assessed with a NanoDrop™ 2000 spectrophotometer (ThermoScientific) and agarose gel electrophoresis. cDNA was synthesized using RevertAid RT Reverse Transcription Kit (ThermoScientific) following the manufacturer’s protocol qPCR was carried out using StepOne™ real-time PCR System (Applied Biosystem, USA) using SYBR® Green Supermix Kit (Bio-Rad, USA). Gene-specific primers were designed using Primer3 software (Version 4.1.0) [52] and listed in Table 1. qRT-PCR assays were carried out by the following conditions mentioned in [53]. 2^-ΔΔCT^ method was used to compare the fold change as described by [54]. The experiment was repeated thrice with three biological and three technical replicates.

**Table 1:** List of Primers used in the experiment

S.N.	Name of Gene	Forward primer 5′-3″	Reverse primer 5′-3′
1.	PR1	5′-TCTGAAGTTGGTGTTGGCCCT-3′	5′-CCGAACCGAATTGCGCCAAA-3′
2.	PDF 1.2	5′-GCTGCCTTGTCCTTCCTCCTCC-3′	5′-AGTGCGCTTTGTTCTTGCAGTG-3′
3.	LOX2	5′-CTGGCCAAAGCTTATGTGGTCGT-3′	5′-TCTCAACGGAATGCTTTGAGGGC-3′
4.	Ubiquitin	5′-CGTTTGAGGGGAGGTATGCAAAT-3′	5′-GTCCTACCATCCTCCAATTGCTTC-3′
5.	NPR1	5′- CTGAGAGAAGGGAGCCTTTACATGG −3′	5′- ACTGCTCTAGACAATGCCTTCATCC − 3′
6.	C4H	5′- TAACCGCCATCACAATCTCA-3′	5′- CTCGACTCCTTGGGTATGGA-3′
7.	NADPH oxidase	5′- GGAGGAGCTTGGACACAGAAGC-3′	5′- TCCACTTCCTCCACTCACCATCA-3′
8.	COI	5′-CGGAGAAACAGATGCAGGGCT-3′	5′-TCTTGGTCCAGCGAGGGAGT-‵3

### Statistical analysis

Statistical analyses were completed using SPSS (Version 24). Experiments were repeated three times using a completely randomized design. Data are expressed as mean of three independent replications ± standard deviation. Treatment means were separated by Duncan’s multiple range test (*P* ≤ 0.05).

## Data Availability

All gene sequences are available through NCBI database.

## Abbreviations

ANE: *Ascophyllum nodosum* extract
PAL: Phenylalanine ammonia lyases
PDI: Percent disease severity
PO: Peroxidase
PPO: Poly phenol oxidase
qRT-PCR: Quantitative real-time polymerase chain reaction
RT: Reverse transcriptase
SD: Standard deviation
TPC: Total phenolic content
